# Performance Drift in Machine Learning Models for Cardiac Surgery Risk Prediction: Retrospective Analysis

**DOI:** 10.2196/45973

**Published:** 2024-06-12

**Authors:** Tim Dong, Shubhra Sinha, Ben Zhai, Daniel Fudulu, Jeremy Chan, Pradeep Narayan, Andy Judge, Massimo Caputo, Arnaldo Dimagli, Umberto Benedetto, Gianni D Angelini

**Affiliations:** 1 Bristol Heart Institute, Translational Health Sciences, University of Bristol, Bristol, United Kingdom; 2 School of Computing Science, Northumbria University, Newcastle upon Tyne, United Kingdom; 3 Department of Cardiac Surgery, Rabindranath Tagore International Institute of Cardiac Sciences, West Bengal, India

**Keywords:** cardiac surgery, artificial intelligence, risk prediction, machine learning, operative mortality, data set drift, performance drift, national data set, adult, data, cardiac, surgery, cardiology, heart, risk, prediction, United Kingdom, mortality, performance, model

## Abstract

**Background:**

The Society of Thoracic Surgeons and European System for Cardiac Operative Risk Evaluation (EuroSCORE) II risk scores are the most commonly used risk prediction models for in-hospital mortality after adult cardiac surgery. However, they are prone to miscalibration over time and poor generalization across data sets; thus, their use remains controversial. Despite increased interest, a gap in understanding the effect of data set drift on the performance of machine learning (ML) over time remains a barrier to its wider use in clinical practice. Data set drift occurs when an ML system underperforms because of a mismatch between the data it was developed from and the data on which it is deployed.

**Objective:**

In this study, we analyzed the extent of performance drift using models built on a large UK cardiac surgery database. The objectives were to (1) rank and assess the extent of performance drift in cardiac surgery risk ML models over time and (2) investigate any potential influence of data set drift and variable importance drift on performance drift.

**Methods:**

We conducted a retrospective analysis of prospectively, routinely gathered data on adult patients undergoing cardiac surgery in the United Kingdom between 2012 and 2019. We temporally split the data 70:30 into a training and validation set and a holdout set. Five novel ML mortality prediction models were developed and assessed, along with EuroSCORE II, for relationships between and within variable importance drift, performance drift, and actual data set drift. Performance was assessed using a consensus metric.

**Results:**

A total of 227,087 adults underwent cardiac surgery during the study period, with a mortality rate of 2.76% (n=6258). There was strong evidence of a decrease in overall performance across all models (*P*<.0001). Extreme gradient boosting (clinical effectiveness metric [CEM] 0.728, 95% CI 0.728-0.729) and random forest (CEM 0.727, 95% CI 0.727-0.728) were the overall best-performing models, both temporally and nontemporally. EuroSCORE II performed the worst across all comparisons. Sharp changes in variable importance and data set drift from October to December 2017, from June to July 2018, and from December 2018 to February 2019 mirrored the effects of performance decrease across models.

**Conclusions:**

All models show a decrease in at least 3 of the 5 individual metrics. CEM and variable importance drift detection demonstrate the limitation of logistic regression methods used for cardiac surgery risk prediction and the effects of data set drift. Future work will be required to determine the interplay between ML models and whether ensemble models could improve on their respective performance advantages.

## Introduction

### Background

Recently, the importance of machine learning (ML), a branch of artificial intelligence, has been highlighted as a potential alternative to traditional mortality risk stratification models such as the Society of Thoracic Surgeons (STS) [1] and European System for Cardiac Operative Risk Evaluation (EuroSCORE) II risk scores [2], which are prone to miscalibration over time and poor generalization across data sets [1,3]. These traditional scoring methods are generally based on logistic regression (LR), with risk factors determined through consensus across experts within leading cardiac surgery organizations in the United States (STS) or Europe (EuroSCORE II). In particular, EuroSCORE II, which is based on LR using 18 items of information about the patient, has been shown by numerous studies to display poor discrimination and calibration across data sets with differing characteristics, including but not limited to age [4], ethnicity [5], and procedures groups [6-10].

Risk scoring models’ performance is challenged by numerous factors, such as differences in variable definitions, the management of incomplete data fields, surgical procedure selection criteria, and temporal changes in the prevalence of patients’ risk factors [11]. ML approaches are increasingly used for prediction in health care research as they have the potential to overcome the limitations of linear models. By including pairwise and higher-order interactions and modeling nonlinear effects, ML may overcome heterogeneity in procedures and missing data [1,12]. Although ML has been shown to be beneficial over conventional scoring systems, the magnitude and clinical influence of such improvements remain uncertain [2]. The ability to counter “performance drift” due to temporal changes in the prevalence of risk factors has also yet to be fully elucidated.

In ML, performance drift refers to the gradual loss in model performance caused by changes that call into question the model’s training assumptions. Key causes of performance drift include data set drift, which refers to changes in the distribution of data between training and evaluation sets; variable importance drift, which involves changes in the significance of model variables; and calibration drift, which is characterized by decreased reliability in estimated probabilities. These factors can interact, as seen in a study of noncardiac surgery [13]. Understanding the complex relationship between variable importance drift, performance drift, and data set drift is important. This relationship explains how changes in the importance of specific variables, combined with changes in the actual data distribution, collectively influence the model’s overall accuracy and reliability as it performs over time. The wider implications are also significant, influencing decision-making, insight accuracy, generalization [14], ethical considerations, and regulatory compliance across industries.

The aim of this study was to investigate performance drift in existing ML models that have been used in prior cardiac surgery risk prediction research. The objectives were to (1) rank and assess the extent of performance drift in such cardiac surgery risk ML models over time and (2) investigate any potential influence of data set drift and variable importance drift on performance drift. Therefore, we trained and evaluated 5 supervised ML models in addition to EuroSCORE II to (1) determine the best ML model in terms of overall accuracy, discrimination, calibration, and clinical effectiveness; (2) use variable importance drift as a measure for detecting data set drift; and (3) verify suspected data set drift informed through variable importance drift by assessing actual data set drift [15].

### Related Work

In our previous study, we found that combining the metrics covering all 4 aspects of discrimination, calibration, clinical usefulness, and overall accuracy into a single clinical effectiveness metric (CEM) improved the efficiency of cognitive decision-making (according to the Miller law [16]) for selecting the optimal ensemble models (ie, using several models to derive a consensus prediction) [14,17]. This approach is useful for providing a consensus metric that enables models to be ranked in scenarios where, for example, 1 model could outperform another using 1 metric but underperform under a different metric. Furthermore, we demonstrated that such a consensus metric could be combined with drill-down analysis to further interpret the models using individual metrics [14]. Although area under the curve (AUC) evaluates the diagnostic or predictive performance of the model, it does not directly reflect patient benefit. This is why we included a suit of other metrics, including the decision curve analysis (DCA) net benefit index, that were found to be clinically pertinent from our prior study [18].

In our previous work [19], we studied the calibration changes across 2 different time intervals using the calibration belt (overall external calibration) and calibration drift (Hosmer-Lemeshow goodness-of-fit *χ*^2^ statistics) approaches within a single UK hospital. A recent study extended our work to a Chinese national registry, Sino (Chinese) System for Coronary Artery Bypass Grafting (CABG) Operative Risk Evaluation II (SinoSCORE II), using a set of ML models such as LightGBM; CatBoost; and a combination of variable selection approaches including Optuna for stepwise regression, BorutaSHAP, and feature importance ranking [20]. Another study in the United States also investigated the calibration performance difference between extreme gradient boosting (XGBoost) and LR models built for a cohort of patients who underwent CABG, using preoperative, intraoperative, and combined variable sets from the STS Adult Cardiac Surgery Database [21].

## Methods

### Data Set and Patient Population

The study was performed using the National Adult Cardiac Surgery Audit (NACSA) data set, which comprises data prospectively collected by the National Institute for Cardiovascular Outcomes Research on all cardiac procedures performed in all National Health Service hospitals and some private hospitals across the United Kingdom [19].

A total of 227,087 adult patients who underwent cardiac surgery between January 1, 2012, and March 31, 2019, were included. Congenital, transplant, and mechanical support device insertion cases were excluded. The CONSORT (Consolidated Standards of Reporting Trials) patient flow diagram is shown in Figure S1 in Multimedia Appendix 1 [19,22-25]. Missing and erroneously inputted data in the data set were cleaned according to the NACSA registry data preprocessing recommendations [26]. Generally, for any variable data that were missing, it was assumed that the variable was at baseline level, that is, no risk factor was present. Missing patient age at the time of surgery was imputed as the median patient age for the corresponding year. Data standardization was performed by subtracting the variable mean and dividing by the SD values [22].

The data set was split into 2 cohorts: training and validation set (n=157,196, 69.2%; 2012-2016; Table S1 in Multimedia Appendix 1) and holdout set (n=69,891, 30.8%; 2017-2019; Table S2 in Multimedia Appendix 1). The primary outcome of this study was in-hospital mortality.

### Baseline Statistical Analysis

Continuous variables were compared using nonparametric Wilcoxon rank sum tests, whereas categorical variables were compared using Pearson *χ*^2^ tests or Fisher exact tests as appropriate.

The *Scikit-learn* (version 0.23.1) and *Keras* (version 2.4.0) Python libraries (Python Software Foundation) were used to develop the models and to evaluate their discrimination, calibration, and clinical effectiveness capabilities. Statistical analyses were conducted using Stata/MP (version 17; StataCorp) and R (version 4.0.2; StataCorp). ANOVA assumptions were checked using the *rstatix* R package.

### Model Development

In our study, we trained 5 supervised ML risk models based on the EuroSCORE II preoperative variable set (Table S3 in Multimedia Appendix 1). Those 5 models included LR, neural network (NN) [22], random forest (RF) [27], weighted support vector machine (SVM) [28], and extreme gradient boosting (XGBoost) [19,29]. The EuroSCORE II score was calculated for baseline comparison. Internal validation was performed using 5-fold cross-validation on the training and validation set (2012-2016) to select model parameters. Final models were determined by retraining the models on the combined training and validation set using the selected model parameters. Temporal external validation was performed using the final models on the holdout set (2017-2019) [15]. Each model calculated the probability of surgical mortality for each patient. Overall, 1000 bootstrap samples were taken for all metrics. Further details on model development can be found in the *Model Specification* section in Multimedia Appendix 1.

### Assessment of Model Performance

The models’ performance was measured across four broad parameters:

1. Discrimination: AUC and *F*_1_-score
2. Clinical utility: DCA net benefit index
3. Calibration: 1 – expected calibration error (ECE)
4. Combination of calibration and discrimination: adjusted Brier score

The AUC performances of all variant models were evaluated, and the receiver operating characteristic (ROC) curves were plotted [30]. As a sensitivity analysis, we calculated the *F*_1_-score, which combines precision and recall without explicitly considering the true negative rate in the performance evaluation [31]. This metric adjusts for the biased effect due to the high proportion of alive outcome samples. The DCA net benefit index was used to test clinical benefit [32]. 1 – ECE was used to determine calibration performance, with higher values being better [33]. A special case of the Brier score (1 – Brier score) without the normalization term was used (adjusted Brier score) [34], with higher values indicating better discrimination and calibration performance.

**Figure 1.**
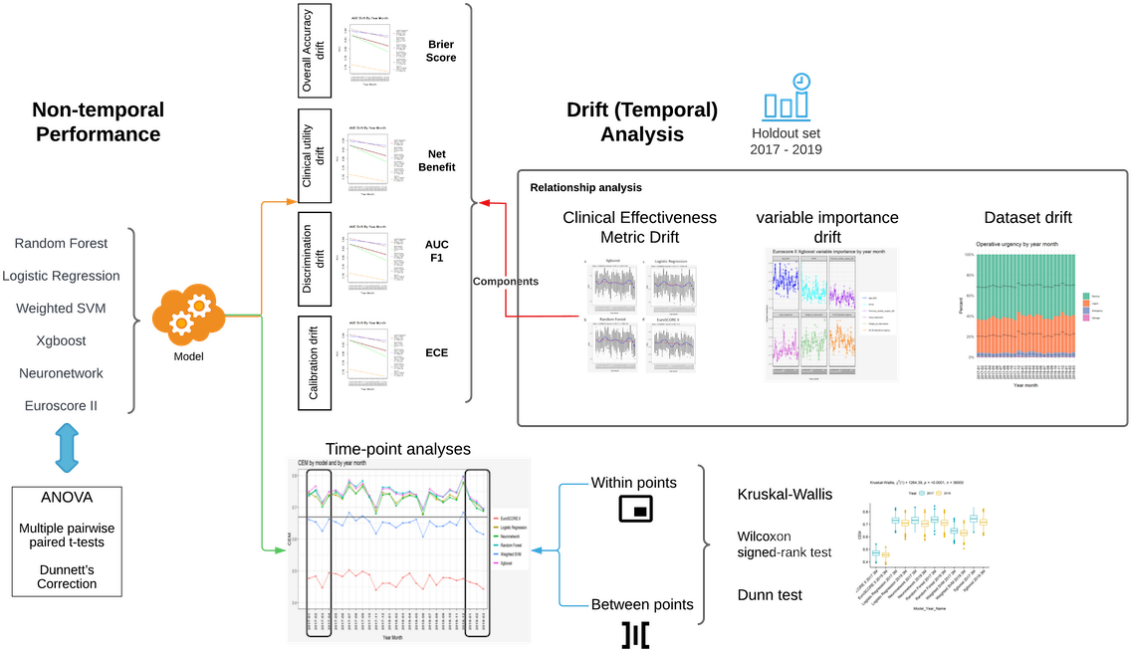
Design overview of the study. Nontemporal performance and drift (temporal) analyses were performed. Drifts in discrimination, calibration, clinical utility, data set, and variable importance were assessed. Time point assessments were performed for the clinical effectiveness metric (CEM). Drifts in component metrics of CEM were evaluated. AUC: area under the curve; ECE: expected calibration error; EuroSCORE: European System for Cardiac Operative Risk Evaluation; F1: *F*_1_-score; neuronetwork: neural network; SVM: support vector machine; Xgboost: extreme gradient boosting.

To determine the best model in terms of both discrimination and calibration, we took the geometric average of AUC, *F*_1_-score [31], DCA net benefit index (treated + untreated), 1 – ECE, and 1 – Brier score. The consensus metric using the combined geometric average of the 5 metrics is named CEM for ease of reference. The consensus approach for combining different metrics has previously been applied in a study on COVID-19 prediction [35]. In addition, this approach is similar to the simple additive weighting multicriteria evaluation approach for making a decision through the ranking of a set of competing criteria [36]. Geometric average has previously been found to be effective for summarizing metrics for temporal-based model calibration and is robust for bootstrap-sampled Gaussian distributions [37]. This metric is robust to outliers [38] and is preferable for aggregation compared to the weighted arithmetic mean [39]. As an exception, the arithmetic average was used for the DCA net benefit index over all thresholds as a measure of overall net benefit, before geometric averaging, since the values can be negative. An overview of the model and evaluation design is shown in Figure 1.

### Baseline Nontemporal Performance

Nontemporal comparison of models was conducted as a baseline, using all data across the holdout period. Differences across models were tested using repeated-measures 1-way ANOVA and Bonferroni-corrected, multiple pairwise, paired *t* tests (1-tailed); this was followed by Dunnett correction for multiple comparisons, with the overall best-performing model as the control. ANOVA assumptions for outliers were checked. Normality assumptions were checked using the Shapiro-Wilk test [40]. The Delong test was applied to determine whether there was a statistically significant difference across the AUCs of the ROC curves for the top 2 best-performing models. A comparison of individual metrics was conducted.

### Drift Analysis

#### Overview

The statistical methods used for analyzing drift is shown in Table 1. More detailed explanations are provided below.

**Table 1.** Summary of statistical methods used for assessing drift.

Objective and statistical tests		General statistical situations	Rationale for choosing test	Assumptions checked
**Nontemporal comparison of models**				
	Repeated-measures 1-way ANOVA	Comparison of multiple groups for differences	Used for comparing means across multiple models	Outliers (ANOVA assumptions) and normality (Shapiro-Wilk test)
	Paired *t* tests (Bonferroni corrected)	Comparison of paired observations between models	To compare specific model pairs simultaneously	—^a^
	Dunnett correction	Control for multiple comparisons	Controls type I error rate in comparing multiple treatments to a control group in 1-way ANOVA	—
	Delong test	Comparison of the AUCs^b^ of 2 correlated ROC^c^ curves	To compare the AUCs of 2 models or tests during sensitivity testing	—
**Analysis within specific time frames**				
	Kruskal-Wallis Test	Comparison of multiple groups for differences (nonparametric)	Nonparametric alternative for ANOVA in specific time frames	Outliers (ANOVA assumptions) and normality (Shapiro-Wilk test)
	Bonferroni-corrected, paired-samples Wilcoxon test (Wilcoxon signed rank test)	Comparison of paired observations within time frames	Nonparametric comparison of paired samples within time frames, with control for type I error rate in comparing multiple treatments	—
	Dunn test	Multiple pairwise comparisons within nonparametric groups	Post hoc test for pairwise comparisons after Kruskal-Wallis test; determines the magnitude of difference effects within time frames	—
**Analysis between the first 3 months of 2017 and 2019**				
	Kruskal-Wallis test	Comparison of multiple groups for differences (nonparametric)	Nonparametric comparison between time frames	Outliers (ANOVA assumptions) and normality (Kolmogorov-Smirnov Test)
	Paired-samples Wilcoxon test (Wilcoxon signed rank test)	Comparison of paired observations between time frames	Nonparametric comparison of paired samples between time frames	—
	Bonferroni-adjusted Dunn test	Multiple pairwise comparisons between time frames	Post hoc test for pairwise comparisons after significant Kruskal-Wallis results; determines the magnitude of difference effects between time frames, with control for type I error rate in comparing multiple treatments	Normality (Kolmogorov-Smirnov Test)
**Analysis of discrimination, calibration, clinical utility, and overall accuracy drift**				
	Linear regression (with residual analysis)	Assessing relationships and regression parameters	To analyze linear relationships and model residuals	Normality through histograms and QQ plots
	Seasonal Kendall test (nonparametric alternative if assumptions not met)	Assessing association or trends when assumptions are not met	Nonparametric test for assessing associations without assumptions	Homoscedasticity through scale-location plots

a Not applicable.

b AUC: area under the curve.

c ROC: receiver operating characteristic.

#### CEM Regression Trends

The geometric CEM mean (and 95% CI) value of 1000 bootstraps for each model against time (the month of the year) was calculated, and the results were plotted to compare trends across models. The models were compared by fitting multiple linear regression lines across time for CEM.

To check for normality assumptions, we plotted the histogram and a QQ plot of residuals before applying linear regressions [41]. We also checked for homogeneity of residual variance (homoscedasticity) by plotting a scale-location plot, that is, the square root of standardized residual points against the values of the fitted outcome variable [42]. For model metrics that do not satisfy these assumptions, the seasonal Kendall test (nonparametric) was used instead.

#### Analysis Within the First 3 Months of 2017 and 2019

Differences in CEM values across models at 2 time points were independently tested using the Kruskal-Wallis test and Bonferroni-corrected, paired-samples Wilcoxon test (Wilcoxon signed rank test). The 2 time points were the first 3 months of 2017 and 2019. This was followed by the Dunn test for nonparametric multiple comparisons of the models at each of the 2 time points, with the overall best-performing model as a baseline. ANOVA assumptions for outliers were checked. Normality assumptions were checked using the Shapiro-Wilk test [40].

#### Analysis Between the First 3 Months of 2017 and 2019

Differences in CEM values across the first 3 months of 2017 and 2019 were tested using the Kruskal-Wallis test and paired-samples Wilcoxon test (Wilcoxon signed rank test). The Bonferroni-adjusted Dunn test was used to determine the magnitude and evidence of change across the 2 time points for each model. ANOVA assumptions for outliers were checked. Normality assumptions were checked using the Kolmogorov-Smirnov Test.

#### Analysis of Discrimination, Calibration, Clinical Utility, and Overall Accuracy Drift

As a sensitivity analysis, we analyzed performance drift in terms of component metrics within CEM. Discrimination (AUC), positive outcome discrimination (*F*_1_-score), calibration (1 – ECE), clinical utility (net benefit), and overall accuracy of prediction probability (adjusted Brier score) were assessed by fitting multiple (model) linear regression lines across time for each metric.

To check for normality assumptions, the same methods as those used for CEM regression trends were used.

### Analysis of Variable Importance Drift

Variable importance drift was assessed for the best-performing model. For each month of the holdout set, 5-fold nested cross-validation was performed to derive the importance of each EuroSCORE II variable in the model’s decision-making. The geometric mean of 5-fold importance at each time point was plotted along with the importance of each of the 5 folds. The Shapley additive explanations (SHAP) mean absolute magnitude of importance was used [43,44]. Locally estimated scatterplot smoothing was used to simplify the visual representation. Line plots of the top 6 most important variables were used as a sensitivity analysis.

### Data Set Drift

Data set drift across time was visualized using a stacked bar plot for the top 3 variables as identified by SHAP variable importance. Continuous variables were binned into intervals to enable ease of analysis.

### Net Benefit Projection

To further understand the clinical significance of the performance drift over time, the fitted linear regression model intercepts and slopes were used to extrapolate the net benefit up to January 2030 for the XGBoost and NN models.

### Ethical Considerations

The study was part of a research project approved by the Health Research Authority and Health and Care Research Wales on July 23, 2019 (Integrated Research Application System project ID: 257758). As the study included retrospective interrogation of the National Institute for Cardiovascular Outcomes Research database, the need for individual patient consent was waived in accordance with the research guidance. The study was performed in accordance with the ethical standards as laid down in the 1964 Declaration of Helsinki and its later amendments.

## Results

### Baseline Patient Characteristics

A total of 227,087 procedures of adults from 42 hospitals were included in this analysis. This followed the removal of 3930 congenital cases, 1586 transplant and mechanical support device insertion cases, and 3395 procedures with missing information on mortality (Table 2). There were 6258 deaths during the study period (mortality rate of 2.76%).

**Table 2.** Patient demographics and summary of cleaned EuroSCORE^a^ II variables. Variables are from the time period from 2012 to 2019. Records with missing mortality status were excluded.

Variable		Mortality status		*P* value^b^
		No (n=220,829)	Yes (n=6258)	
Age (years), mean (SD)		67.53 (11.23)	70.77 (11.42)	<.001
**NYHA^c^ classification, n (%)**				<.001
	0 (I)	48,625 (22)	1055 (17)	
	1 (II)	96,888 (44)	1609 (26)	
	2 (III)	64,049 (29)	2228 (36)	
	3 (IV)	11,267 (5.1)	1366 (22)	
**Renal impairment, n (%)**				<.001
	0 (normal)	103,196 (47)	1704 (27)	
	1 (moderate)	92,411 (42)	2451 (39)	
	2 (on dialysis)	2187 (1)	330 (5.3)	
	3 (severe)	23,035 (10)	1773 (28)	
Chronic lung disease, n (%)		26,644 (12)	1211 (19)	<.001
Poor mobility, n (%)		8305 (3.8)	514 (8.2)	<.001
Previous cardiac surgery, n (%)		12,012 (5.4)	1141 (18)	<.001
**Left ventricle function, n (%)**				<.001
	0 (good; >50%)	184,721 (84)	4706 (75)	
	1 (moderate; 31%-50%)	30,608 (14)	1089 (17)	
	2 (poor; 21%-30%)	4241 (1.9)	318 (5.1)	
	3 (very poor; ≤20%)	1259 (0.6)	145 (2.3)	
**Pulmonary hypertension, n (%)**				<.001
	0 (PA^d^ systolic <31 mm Hg)	201,643 (91)	5000 (80)	
	1 (PA systolic 31-55 mm Hg)	13,126 (5.9)	705 (11)	
	2 (PA systolic >55 mm Hg)	6060 (2.7)	553 (8.8)	
CCS^e^ class 4 angina, n (%)		18,370 (8.3)	956 (15)	<.001
**Urgency, n (%)**				<.001
	0 (elective)	141,617 (64)	2442 (39)	
	1 (urgent)	72,090 (33)	2134 (34)	
	2 (emergency)	6533 (3)	1230 (20)	
	3 (salvage)	589 (0.3)	452 (7.2)	
**Weight of the intervention, n (%)**				<.001
	0 (isolated CABG^f^)	111,243 (50)	1546 (25)	
	1 (single non-CABG)	62,568 (28)	2153 (34)	
	2 (two procedures)	42,649 (19)	2108 (34)	
	3 (three procedures)	4369 (2)	451 (7.2)	
Diabetes on insulin, n (%)		12,818 (5.8)	453 (7.2)	<.001
Female gender, n (%)		59,467 (27)	2328 (37)	<.001
Recent myocardial infarction, n (%)		43,316 (20)	1594 (25)	<.001
Critical preoperative state, n (%)		7255 (3.3)	1382 (22)	<.001
Extracardiac arteriopathy, n (%)		22,327 (10)	1215 (19)	<.001
Active endocarditis, n (%)		5816 (2.6)	493 (7.9)	<.001
Surgery on thoracic aorta, n (%)		9070 (4.1)	896 (14)	<.001
EuroSCORE II, mean (SD)		0.03 (0.04)	0.12 (0.14)	<.001

a EuroSCORE: European System for Cardiac Operative Risk Evaluation.

b Wilcoxon rank sum test or Pearson *χ*2 test

c NYHA: New York Heart Association.

d PA: pulmonary artery.

e CCS: Canadian Cardiovascular Society.

f CABG: coronary artery bypass grafting.

### Baseline Nontemporal Performance

No extreme outliers were found when testing for ANOVA assumptions. The CEM values from 1000 bootstraps were normally distributed for LR, NN, and RF but not XGBoost, as assessed by the Shapiro-Wilk test (*P*>.05). A histogram plot of the XGBoost CEM values did not show substantial deviation from the normal distribution. There was strong evidence of a difference across all models (*P*<.0001; Table S4 and Figure S2 in Multimedia Appendix 1). Table 3 shows that XGBoost (CEM 0.728, 95% CI 0.728-0.729) and RF (CEM 0.727, 95% CI 0.727-0.728) were the overall best-performing models, with moderate to strong evidence (nonoverlapping CIs) of the former outperforming the latter. This was followed by LR, NN, SVM, and EuroSCORE II. The Dunnett test showed that there was moderate to strong evidence that XGBoost was superior to all other models (*P*<.001; Table 4). The performance of XGBoost was the least different from RF but the most different from EuroSCORE II (CEM difference to XGBoost: 0.0009 vs 0.1876).

The sensitivity analysis of CEM component metrics showed that the adjusted Brier score was unable to distinguish between XGBoost, RF, NN, and LR (Table 3; all 0.976). AUC performance was the best for XGBoost (0.834) and RF (0.835), with the Delong test showing no statistically significant difference (*P*>.05). *F*_1_-score showed that XGBoost performed the best, followed by RF (0.279 vs 0.277). LR and NN (adjusted ECE: both 0.997) showed better calibration performance than RF and XGBoost (adjusted ECE: both 0.996). Net benefit was the best for XGBoost and RF (both 0.904).

**Table 3.** Geometric mean of individual metrics for each model in the holdout set. In all, 1000 bootstrap samples were used to derive the geometric mean of each metric. Adjusted ECE^a^ and Brier score values are shown. Net benefit is the average absolute overall benefit across all thresholds.

Model category	1 – ECE	AUC^b^	1 – Brier score	*F*_1_-score	Net benefit	CEM^c^				
						Mean (SD)		95% CI		Value, n
EuroSCORE^d^ II	0.641	0.800	0.814	0.240	0.461	0.541 (0.004)		0.540-0.541		1000
LR^e^	0.997	0.819	0.976	0.264	0.902	0.717 (0.005)		0.717-0.717		1000
NN^f^	0.997	0.813	0.976	0.259	0.901	0.713 (0.006)		0.713-0.714		1000
RF^g^	0.996	0.835	0.976	0.277	0.904	0.727 (0.005)		0.727-0.728		1000
Weighted SVM^h^	0.775	0.819	0.916	0.257	0.685	0.634 (0.005)		0.634-0.634		1000
XGBoost^i^	0.996	0.834	0.976	0.279	0.904	0.728 (0.005)		0.728-0.729		1000

a ECE: expected calibration error.

b AUC: area under the curve.

c CEM: clinical effectiveness metric.

d EuroSCORE: European System for Cardiac Operative Risk Evaluation.

e LR: logistic regression.

f NN: neural network.

g RF: random forest.

h SVM: support vector machine.

i XGBoost: extreme gradient boosting.

**Table 4.** The Dunnett test with XGBoost^a^ as a control and the rest of the models as comparisons.

Group 1	Group 2 (control)	CEM^b^ difference (group 1 – group 2; 95% family-wise CI)	*P* value
EuroSCORE^c^ II	XGBoost	−0.1876 (−0.1881 to −0.1870)	<2×10^–16^^d^
LR^e^	XGBoost	−0.0110 (−0.0116 to −0.0105)	<2×10^–16^^d^
NN^f^	XGBoost	−0.0148 (−0.0154 to −0.0142)	<2×10^–16^^d^
RF^g^	XGBoost	−0.0009 (−0.0015 to −0.0003)	.00039^d^
Weighted SVM^h^	XGBoost	−0.0941 (−0.0947 to −0.0935)	<2×10^–16^^d^

a XGBoost: extreme gradient boosting.

b CEM: clinical effectiveness metric.

c EuroSCORE: European System for Cardiac Operative Risk Evaluation.

d *P*<.001.

e LR: logistic regression.

f NN: neural network.

g RF: random forest.

h SVM: support vector machine.

### Drift Analysis

#### Overall CEM

**Figure 2.**
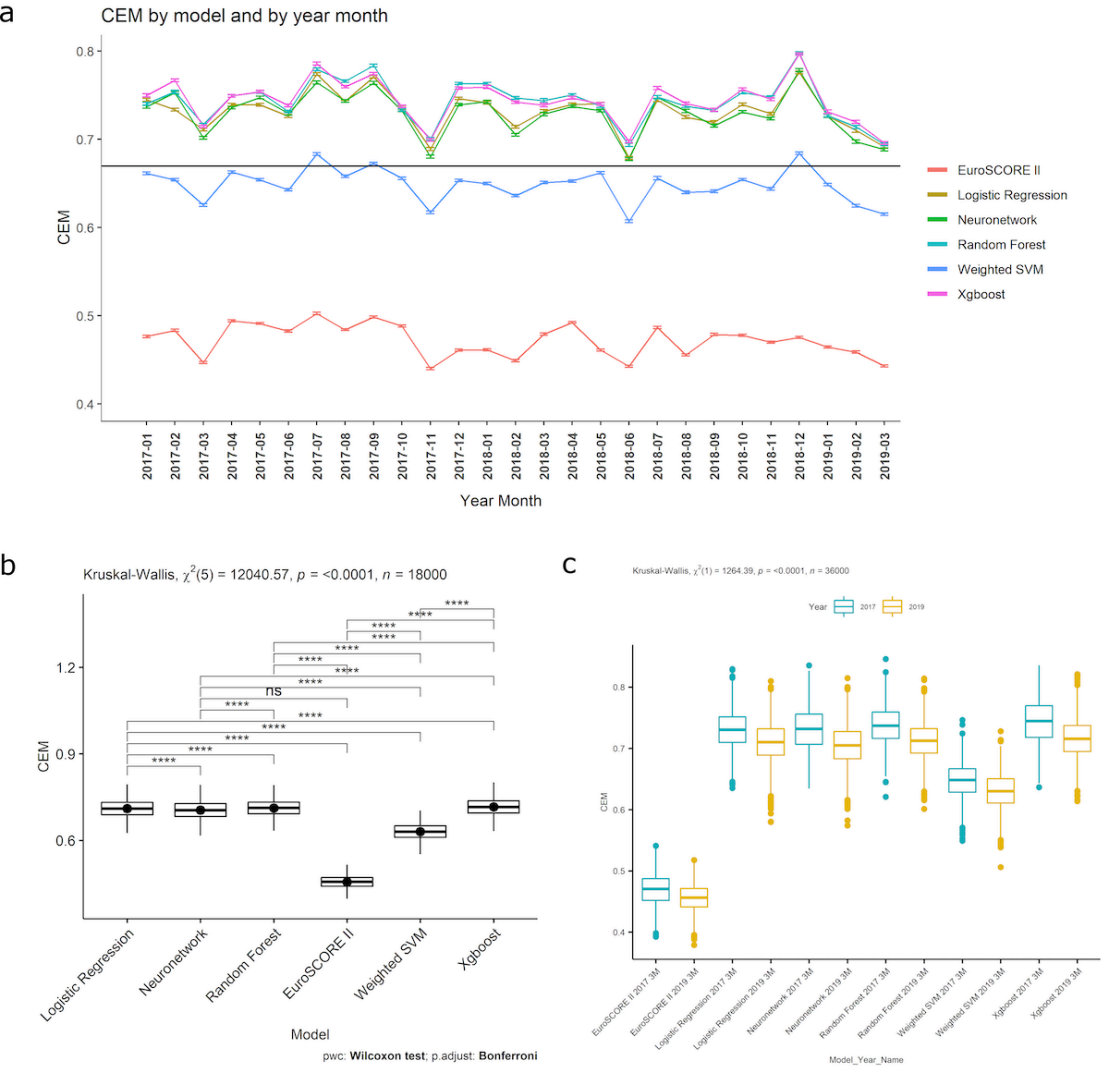
(A) Plot of CEM values by model and time. Geometric mean (95% CI) of 1000 bootstraps at each time point is shown. The horizontal line represents the CEM geometric mean of all models. (B) Box plot of difference in models’ CEM values across the first 3 months of 2017 and 2019. Kruskal-Wallis results for CEM across the time points are shown. (C) Paired-samples Wilcoxon test (Wilcoxon signed rank test) for the first 3 months of 2019 bootstrap CEM values. *P* values are adjusted using the Bonferroni method. ****P<.0001. CEM: clinical effectiveness metric; EuroSCORE: European System for Cardiac Operative Risk Evaluation; ns: not significant; neuronetwork: neural network; SVM: support vector machine; Xgboost: extreme gradient boosting.

Figure 2A shows that XGBoost and RF were candidates for the best overall CEM performance across time. There was minor evidence of LR outperforming NN across time. Seasonal fluctuations were observed. EuroSCORE II performed the worst across time, followed by SVM.

**Figure 3.**
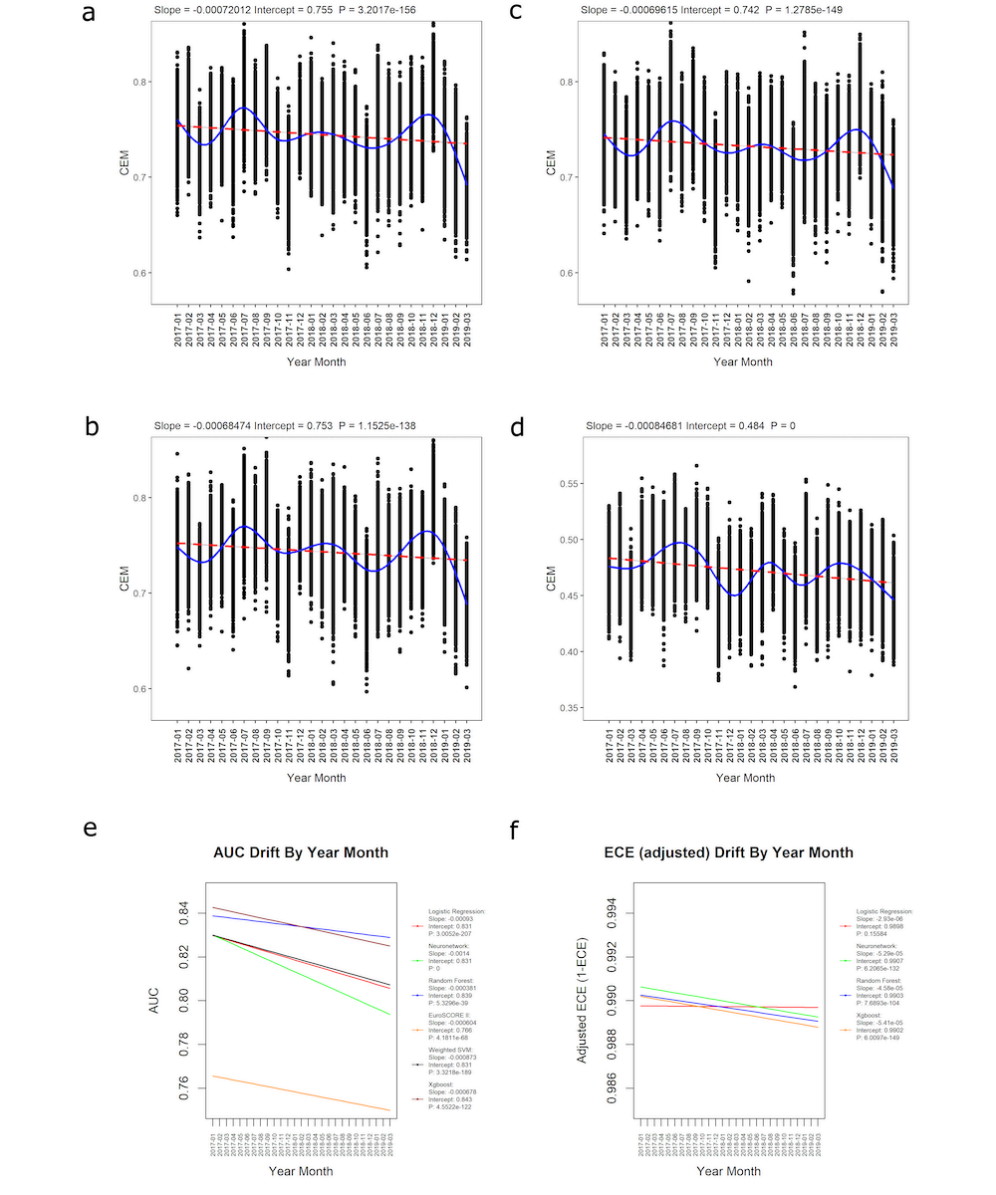
Plots of CEM values by model and time: (A) XGBoost, (B) random forest, (C) logistic regression, and (D) EuroSCORE II. The geometric mean of 1000 bootstraps at each time point is shown. The red dotted line shows linear regression, and the blue line shows generalized additive model fit. Parameters and *P* values for the linear regressions are shown. (E) Discrimination (AUC) performance drift by time. Linear regression lines are plotted for each model, with slope, intercept, and *P* values displayed in the legend. (F) Calibration (adjusted ECE) performance drift by time. Linear regression lines are plotted for each model, with slope, intercept and *P* values displayed in the legend. SVM and EuroSCORE II are removed to enable a clearer separation of models with similar performance. AUC: area under the curve; CEM: clinical effectiveness metric; ECE: expected calibration error; EuroSCORE: European System for Cardiac Operative Risk Evaluation; neuronetwork: neural network; SVM: support vector machine; Xgboost: extreme gradient boosting.

There was strong evidence of a decrease in overall performance across all models (*P*<.0001). Linear regression plots showed that XGBoost had the best starting CEM (intercept: 0.755 vs 0.753 [RF], 0.742 [LR], and 0.741 [NN]), but the rate of performance decrease (slope −0.000720) was less than NN (−0.00083) and greater than RF (−0.000685) and LR (−0.000696; Figure 3A-C and Figure S3 in Multimedia Appendix 1). By March 2019, the overall CEM performance ranking was not changed, with XGBoost performing the best, followed by RF, LR, and NN. EuroSCORE II (intercept 0.484; slope −0.000847) performed the worst in terms of starting CEM and rate of performance decrease, followed by SVM (intercept 0.658; slope −0.000625; Figure 3D and Figure S4 in Multimedia Appendix 1). Normality and homogeneity assumptions were satisfied for all models’ CEM values, as checked by a QQ plot of residuals and scale-location plot (Figure S5 in Multimedia Appendix 1).

#### Analysis Within the First 3 Months of 2017

No extreme outliers were found for the models’ CEM values in the first 3 months of 2017. The CEM values were nonnormally distributed for all models (*P*<.05; Table S5 in Multimedia Appendix 1). There was strong evidence of a difference across all models (*P*<.0001; Table 3 and Figure S6 in Multimedia Appendix 1). The Dunn test showed strong evidence of XGBoost having the best overall performance (Table S6 in Multimedia Appendix 1; *P*<.0001), followed by RF, NN, and LR (CEM difference to XGBoost: −0.0076, −0.0124, and −0.0138, respectively; *P*<.0001). EuroSCORE II performed the worst, followed by weighted SVM (CEM difference to XGBoost: −0.2739 and −0.0961, respectively; *P*<.0001).

#### Analysis Within the First 3 Months of 2019

No extreme outliers were found for the models’ CEM values in the first 3 months of 2019. The CEM values were nonnormally distributed for 50% (3/6) of models (*P*<.05). There was strong evidence of a difference across all models (*P*<.0001; Table S7 in Multimedia Appendix 1 and Figure 2B). The Dunn test showed strong evidence of XGBoost having the best overall performance (Table S8 in Multimedia Appendix 1; *P*<.05), followed by RF, LR, and NN (CEM difference to XGBoost: −0.0032, −0.0055, and −0.0108, respectively; *P*<.05). EuroSCORE II performed the worst, followed by weighted SVM (CEM difference to XGBoost: −0.2594 and −0.0856, respectively; *P*<.0001).

#### Analysis Between the First 3 Months of 2017 and 2019

No extreme outliers were found for the models’ CEM values in the first 3 months of 2017 and 2019. The CEM values were nonnormally distributed for the first 3 months of 2017 and 2019, as assessed by the Kolmogorov-Smirnov test (*P*<.05). There was strong evidence of an overall difference across the 2 time points (*P*<.0001; Table S9 and Figure S7 in Multimedia Appendix 1). There was strong evidence of a difference across the 2 time points for each individual model (*P*<.05; Figure 2C and Table S10 in Multimedia Appendix 1). XGBoost retained the best overall performance across the time points examined. This model showed the largest decrease in CEM performance (median difference 0.0288; *P*<.0001), followed by NN, RF, and LR (median difference: 0.0272, 0.0244, and 0.0205, respectively; *P*<.0001). Following a performance decrease from 2017 to 2019, XGBoost still had the best overall performance, with RF being the second best (median CEM: 0.716 and 0.713, respectively). Although NN had a better starting performance than LR, the larger performance drift resulted in NN having a lower overall performance than LR in 2019 (median CEM: 0.705 vs 0.710). Although the performance drift was smaller, LR’s CEM performance never exceeded RF’s (median CEM: 0.710 vs 0.713). EuroSCORE II showed the least performance drift, followed by weighted SVM (median difference: 0.0142 and 0.0183, respectively; *P*<.05), but both performed the worst in terms of absolute CEM value.

#### Analysis of Discrimination, Calibration, and Clinical Effectiveness Drift

##### Discrimination

###### AUC

Linear regression plots showed that XGBoost had the best starting AUC (intercept: 0.843 vs 0.839 [RF] and 0.831 [LR, NN, and SVM]), but the rate of performance decrease was greater than RF and EuroSCORE II (slope: −0.000678 vs −0.000381 [RF] and −0.000604 [EuroSCORE II]; Figure 3E). By March 2019, XGBoost’s AUC had decreased below RF’s, resulting in RF being the best-performing model, followed by XGBoost, SVM, LR, and NN. NN showed the largest rate of AUC decrease, followed by LR and SVM (slope: −0.0014, −0.00093, and −0.000873, respectively). EuroSCORE II performed the worst in terms of AUC across all time points (intercept 0.766). There was strong evidence of a decrease in AUC performance across all models (*P*<.0001). Normality and homogeneity assumptions were satisfied for all models’ AUC values, as checked by a QQ plot of residuals and scale-location plot (Figure S8 in Multimedia Appendix 1).

###### *F*_1_-score

The best-performing model across all holdout time periods was XGBoost, followed by RF, LR, NN, SVM, and EuroSCORE II. There was strong evidence of a decrease in *F*_1_-score performance across all models (*P*<.0001). More details can be found in the *Positive Outcome Discrimination* section and Figures S9-10 in Multimedia Appendix 1.

### Calibration

Linear regression plots showed that NN has the best starting adjusted ECE (intercept: 0.9907 vs 0.9903 [RF], 0.9902 [XGBoost], and 0.9898 [LR]), but the rate of performance decrease was greater than LR and RF (slope: −5.29×10^–5^ vs −2.93×10^–6^ [LR] and −4.58×10^–5^ [RF]; Figure 3F). By March 2019, NN’s adjusted ECE had decreased below LR’s, resulting in LR being the best-performing model, followed by NN, RF, and XGBoost. Although SVM and EuroSCORE II had lower rates of adjusted ECE decrease (slope: −0.000251 and −0.000479, respectively), the calibration performance was much lower at all time points compared to the other models (Figure S11 in Multimedia Appendix 1). There was strong evidence of a decrease in adjusted ECE performance across all models (*P*<.0001), except LR (*P*>.05). Normality and homogeneity assumptions were satisfied for all models’ adjusted ECE values, as checked by a QQ plot of residuals and scale-location plot (Figure S12 in Multimedia Appendix 1).

### Clinical Effectiveness

**Figure 4.**
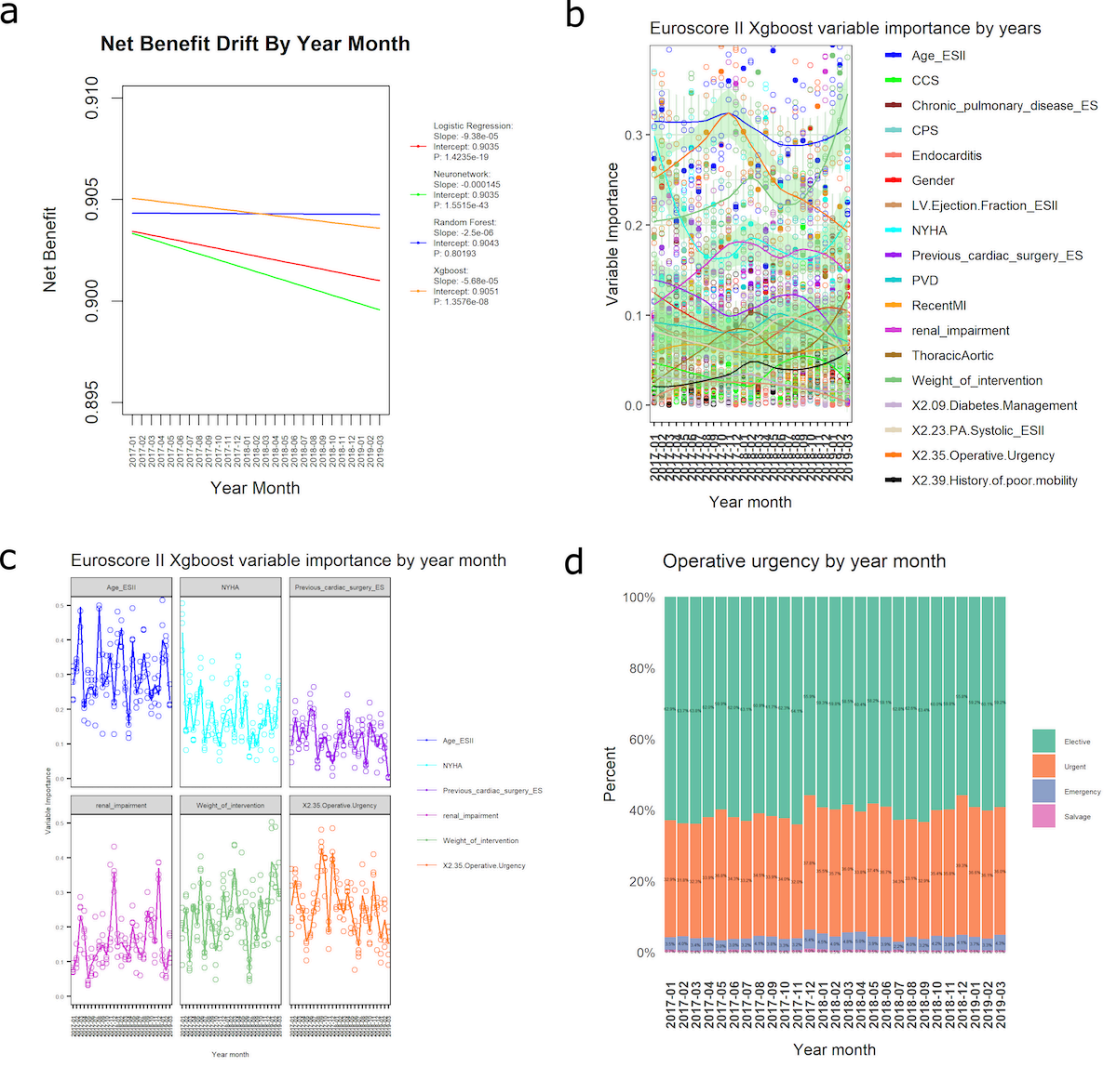
(A) Clinical effectiveness (net benefit) performance drift by time. Linear regression lines are plotted for each model, with slope, intercept, and *P* values displayed in the legend. SVM and EuroSCORE II are removed to enable a clearer separation of models with similar performance. (B) SHAP variable importance drift for the holdout set over 27 months (EuroSCORE II and XGBoost). Solid dots show geometric mean values of 5-fold cross-validation. Smoothed locally estimated scatterplot lines are plotted, with green bands showing 95% CIs. (C) SHAP variable importance drift for the holdout set over 27 months for the top 6 most important variables (EuroSCORE II and XGBoost). The trends are unsmoothed. (D) Operative urgency data set drift across time for the holdout set. The percentages of each category are shown for each time point. CCS: Canadian Cardiovascular Society; CPS: critical preoperative state; EuroSCORE: European System for Cardiac Operative Risk Evaluation; ES: EuroSCORE; LV: left ventricle; MI: myocardial infarction; neuronetwork: neural network; NYHA: New York Heart Association; PA: pulmonary artery; PVD: peripheral vascular disease; SHAP: Shapley additive explanations; SVM: support vector machine; Xgboost: extreme gradient boosting.

Linear regression plots showed that XGBoost had the best starting net benefit (intercept: 0.9051 vs 0.9043 [RF] and 0.9035 [NN and LR]), but the rate of performance decrease was greater than RF (slope: −5.68×10^–5^ vs −2.5×10^–6^; Figure 4A), slower than LR (−9.38×10^–5^), and even slower than NN (−0.000145). By March 2019, XGBoost’s net benefit had decreased below RF’s, resulting in RF being the best-performing model, followed by XGBoost, LR, and NN. EuroSCORE II showed the largest rate of net benefit decrease and performed the worst across all time points, followed by SVM (intercept: 0.314 and 0.690; slope: −0.000846 and −0.000364, respectively; Figure S13 in Multimedia Appendix 1). There was strong evidence of a decrease in net benefit performance across all models (*P*<.0001), except RF (*P*>.05). Normality and homogeneity assumptions were satisfied for all models’ net benefit values, as checked by a QQ plot of residuals and scale-location plot (Figure S14 in Multimedia Appendix 1).

### Accuracy of Prediction Probability

By March 2019, XGBoost was the best model, followed by RF, LR, and NN. EuroSCORE II performed the worst in terms of adjusted Brier score and rate of decrease, followed by SVM. There was strong evidence of a decrease in adjusted Brier score performance across all models (*P*<.0001), except XGBoost and RF. More details can be found in the *Accuracy of Prediction Probability* section and Figures S15-S17 in Multimedia Appendix 1.

### Analysis of Variable Importance Drift

SHAP mean absolute magnitude of importance was used to measure variable importance drift for the best temporal and nontemporal model (XGBoost). Smoothed trend lines showed substantial drift in numerous variables, including the most important variables: age, operative urgency, the weight of intervention, New York Heart Association classification, renal impairment, and previous cardiac surgery (Figure 4B). The sensitivity analysis showed a substantial drift in variable importance across the holdout set for all 6 variables (Figure 4C). When compared with the CEM performance drop from October to December 2017 and from June to July 2018 (Figure 3 generalized additive model line), it could be seen that the CEM decrease was mirrored by decreases in the importance of the top variables, age and operative urgency, at these time periods (Figure 4C). A decrease in CEM performance in the 3 months of 2019 was likely to be at least partly contributed by the sudden rise in the importance of the weight of intervention (Figure 3 and Figure 4B and C).

### Data Set Drift Across Time

Data set drift was observed throughout the holdout time periods for operative urgency, with sharp drifts observed across all categories from November to December 2017 and from June to July 2018 (Figure 4D). Data set drift was observed across the holdout time periods for the <60 and >60 years patient age groups (Figure S18 in Multimedia Appendix 1), with marked data drifts observed from October to November 2017 and from July to August 2018. Data set drift was observed across the holdout time periods for the weight of intervention (Figure S19 in Multimedia Appendix 1). Sharp data set drifts were observed for the single non-CABG and 3 procedures categories from December 2018 to February 2019.

### Net Benefit Projection

**Figure 5.**
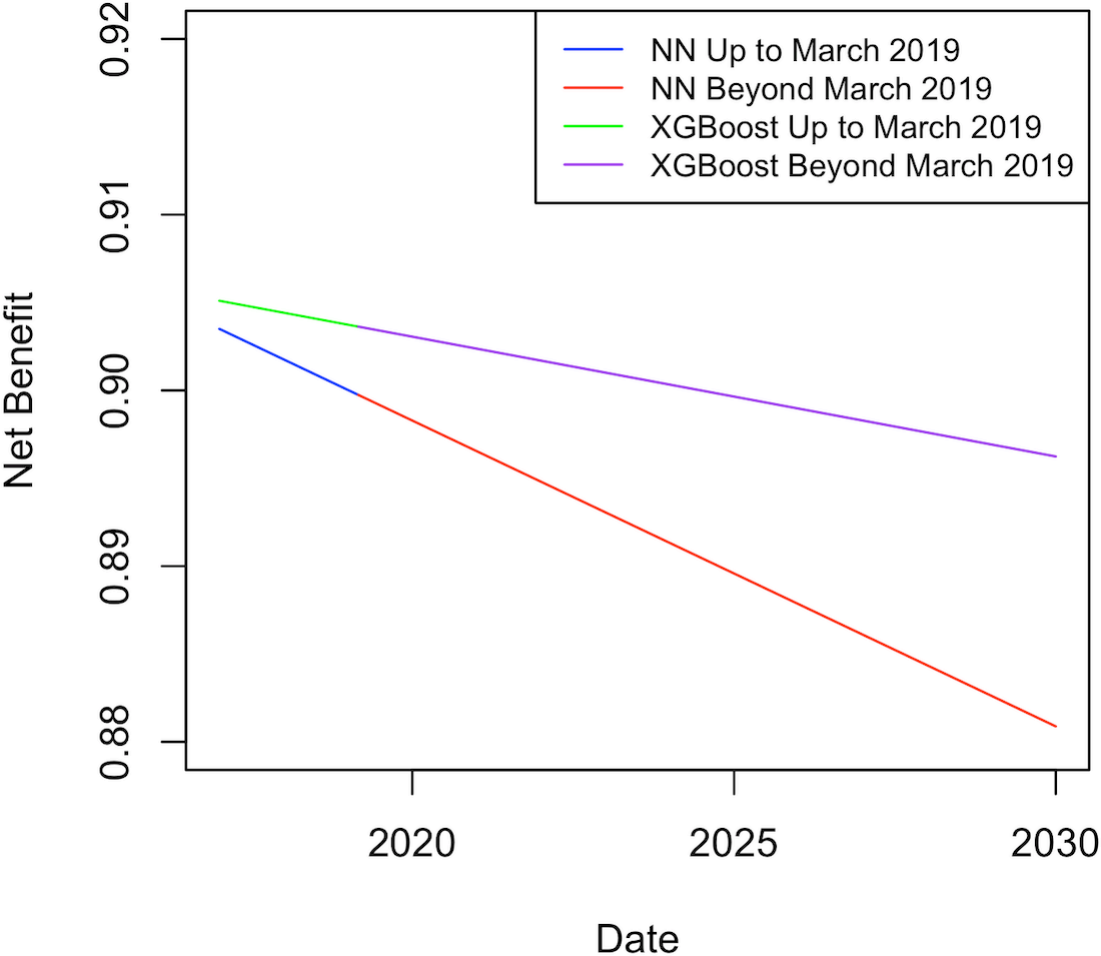
The actual and projected net benefit drift for the NN and Xgboost models over time. NN: neural network; XGBoost: extreme gradient boosting.

To further understand the clinical significance of performance drift over time, Figure 5 illustrates the expected net benefit decrease for the NN and XGBoost models. The blue line depicts the actual net benefit drop for the NN model (as represented by the slope), transitioning to the projected red line after March 2019. The green line represents the actual net benefit drop for the XGBoost model up to March 2019, changing to the projected purple line after March 2019. A clinically significant decrease (from 0.9035 to 0.8808) is shown for NN but not for XGBoost (from 0.9051 to 0.8962).

## Discussion

### Principal Findings

The main finding of the study was that the XGBoost model performed the best, followed by RF, LR, and NN, when all metrics were simultaneously considered, both temporally and nontemporally. Furthermore, EuroSCORE II substantially underperformed against all ML models across all comparisons; this presents an urgent need to understand the drift effects of this score and is not limited to calibration drift. By first combining all metrics and then analyzing the temporal drift of each metric individually, we were able to determine the contribution of individual metrics to the overall performance drift of each model. We found strong evidence that all models showed a decrease in at least 3 of the 5 individual metrics within the CEM. This demonstrated the importance for clinicians and ML governance teams to actively monitor the effects of data set drift (as explained later) on “big data” models that are prepared for or being clinically used to minimize the risk of harm to patients.

“Big data” refers to large and detailed data sets that are suited to ML analyses rather than traditional statistical analyses [45,46], and they are increasingly used in health care. These analyses can inform, personalize, and potentially improve care [45,47,48]. Despite growing interest [49] in ML and health care data linkage initiatives such as the Cardiac Quality Assurance Programme in the United Kingdom [50], there have been limited reports of use within cardiac surgery [51-53], with one of the main reasons being a lack of understanding by clinicians of the underlining processes [54].

As more countries follow in the steps of the United States to deploy ML to the medical settings [55], it becomes increasingly critical that clinicians and ML governance teams are adequately prepared for situations in which ML systems fail to perform their intended functions [56]. A major factor in ML malfunction is “data set drift,” where ML performance declines due to a mismatch between the data on which the model was trained and the new unseen data to which the model is applied [57]. Several factors have been reported to influence data set drift, including changes in technology, demographics, and patient or clinician behavior [56].

In our previous systematic review, we found that despite ML models achieving better discriminatory ability than traditional LR approaches, few cardiac surgery studies assessed calibration, clinical utility, discrimination, and data set drift collectively; these aspects should be assessed to determine the clinical implications of ML [2]. Our previous study [19], although not involving the assessment of XGBoost, had also shown that the calibration drift of LR was less than that of RF, whereas EuroSCORE I, naïve Bayes, and NN performed poorly in terms of calibration. A recent study extending on our work had shown temporal and spatial calibration drift (comparison across regions and hospitals) to be severe across a range of ML models using a national Chinese registry [20]. In accordance with our view, the study highlighted that “future efforts may need to shift more towards enhancing model calibration robustness or recalibration for greater practical value” and that the inclusion of intraoperative variables may be important to enhancing model performance. The STS Adult Cardiac Surgery Database study [21] had shown that the inclusion of intraoperative variables improved both the discrimination and calibration performance of XGBoost and LR models in patients who underwent CABG from the United States. Although calibration drift over time is well documented among EuroSCORE and LR models for hospital mortality, the susceptibility of competing ML modeling methods to data set drift has not been well studied in cardiac surgery [13].

This study heeds the call for additional metrics to address the lack of sensitivity of the most commonly used C-statistic and calibration slope in capturing the advantage of ML models [58]; we demonstrated the use of a consensus score [22,35,59-61] named CEM to take into account numerous metrics that have been found to be beneficial, covering overall accuracy [58], discrimination, calibration, and clinical utility. We wanted to analyze model performance across multiple metrics across time in this study.

This study showed invariance in model ranking for the CEM in both temporal and nontemporal analyses, indicating that there is value for this consensus scoring approach in performance drift evaluation.

This study also addresses the gap in understanding the effect of data set drift on the performance of ML and traditional models over time, which presents a barrier to their clinical application. The shift between XGBoost and RF having the best performance for AUC and net benefit and between NN and LR having the best performance for “adjusted ECE” demonstrates that the comparison of models at a single time point was insufficient to understand the clinical limitations of ML models and that at least 2 time points should be considered.

Our study has also found that although RF showed comparable discrimination (AUC) and clinical utility (net benefit) performance across time, the reason for XGBoost’s superior overall temporal performance was in its better overall accuracy (adjusted Brier score) and positive outcome discrimination (*F*_1_-score). *F*_1_-score is often overlooked but is especially important in cardiac surgery data sets, whereby the incidence for the outcome of interest is typically very low and introduces bias in the performance evaluation when AUC is used. We found that RF performed the second best overall. Unlike XGBoost, RF performed better in terms of resistance to drift for AUC and net benefit, suggesting that further work is required to determine whether the synergistic (ensemble) effects across models are beneficial for improving cardiac surgery risk prediction.

Although XGBoost is currently the best temporal and nontemporal model for the NACSA data set, periodic monitoring of performance drift for each yearly revision of this data set should be mandated to determine whether or not performance has been overtaken by RF, and if so, at what point in time this happens [56]. As all models showed strong evidence of a decrease in overall performance from January 2017 to March 2019, further work will be required to develop either better-performing models or models that are less susceptible to performance drift. However, through projecting the net benefit into the year 2030 based on the fitted linear regression, the decreases in the net benefit for XGBoost over time were shown to be clinically insignificant. On the contrary, the NN model showed a clinically significant drop in net benefit.

Although the reported decreases in measures such as CEM and AUC may appear small, such changes are likely to impact the potential use of ML models within clinical scenarios. If such models are to be used clinically for making decisions about the patient, even small changes in these metrics (which have been previously discussed [18] to be important in cardiac surgery ML performance) can have an influence on risk assessment and patient outcomes, necessitating constant model drift monitoring. Prior research has shown that improving model calibration robustness or recalibration is necessary for practical value and that the “the significant decline in performance of previously established models in this study calls for continuing model updates” [20]. It is envisaged that collaboration between physicians and ML scientists is critical. Before mandating model updates, it is critical to establish metric-specific thresholds for acceptable reductions. A consensus approach, extensive experience in this area, or a meta-analysis of current literature may be required for this collaborative decision-making process.

We have demonstrated that by associating relationships between smoothed [62] and unsmoothed trend lines for CEM performance and EuroSCORE II variable importance, it was possible to detect subtle data set drifts that could result in model performance drifts. Our findings of variable importance and data set drift from October to December 2017, from June to July 2018, and from December 2018 to February 2019 are likely to reflect seasonality changes and mirrored effects of sharp drifts in CEM performance across models. The detection of data set drift was verified by checking for actual drifts in the data set variables. A noncardiac surgery study used actual data set drift to check for variable importance–detected data set drift [13]. However, drift in the actual data set was only analyzed across 2 data points [13], without consideration for smoothed and unsmoothed relationships across performance, variable importance, and actual variable incidence. This study provides the foundations for which further work analyzing ML performance drift are recommended, to analyze relationships between drifts in a consensus score such as CEM and in variable importance, followed by the confirmation of any detected drifts using actual data set trends (data set drift).

### Limitations and Future Studies

Although statistical rigor has been applied to determine whether performance drift is a barrier to clinical risk modeling and decision-making, further work could be done to apply more statistically sensitive approaches for comparing the interactions of trends in data set drift, performance drift, and variable importance drift. As NACSA patient identifiers and the Hospital Episode Statistics data set were not available for linkage, it was not possible to determine whether there were any same patient individuals in both the training and validation set and holdout set, where they had multiple surgeries. Clinical judgment suggests that the proportion of multiple surgeries would be very low. Nonetheless, future work should consider the collection of such information to minimize any potential bias. Our previous work using CEM and constituent metrics to study random effects ML had also shown that hospital-related systematic variations may be better adjusted for by including hospital location variables as part of the input covariates rather than specifically using mixed effects ML models [17]. Future work may consider the incorporation of such systematic variation adjustments when studying drift effects to further investigate the optimal approach for modeling drift across individual hospitals. Although CEM is a consensus score that enhances the clinical evaluation of complex relationships across different aspects of model performance, compressing the net benefit measure into a single value would mean that further DCA may be required if individual-specific, threshold-based decisions were to be fully considered. Future studies should also delve deeper into the relationships of the studied drift types with concept drift in cardiac surgery risk prediction.

### Conclusion

This study found that performance drift of ML and EuroSCORE II over time could be explained through data set drift patterns in cardiac surgery risk prediction. It was also found that variable importance drift could help to explain performance drift and support the detection of data set drift in the assessed models. The strong evidence of all models showing a decrease in at least 3 of the 5 individual metrics within CEM demonstrates the potential need to update the models over time, but future work are required to determine suitable thresholds for mandating an update. Future work will be required to determine the interplay between XGBoost and RF, which have demonstrated less drift over time, and whether combining these models through additional ensemble modeling could take advantage of their respective performance advantages.

## Supplementary material

10.2196/45973 Multimedia Appendix 1 Data set split, model specification, drift analysis, and other analyses.

## Abbreviations

AUC: area under the curve
CABG: coronary artery bypass grafting
CEM: clinical effectiveness metric
CONSORT: Consolidated Standards of Reporting Trials
DCA: decision curve analysis
ECE: expected calibration error
EuroSCORE: European System for Cardiac Operative Risk Evaluation
LR: logistic regression
ML: machine learning
NACSA: National Adult Cardiac Surgery Audit
NN: neural network
RF: random forest
ROC: receiver operating characteristic
SHAP: Shapley additive explanations
SinoSCORE II: Sino (Chinese) System for Coronary Artery Bypass Grafting Operative Risk Evaluation II
STS: Society of Thoracic Surgeons
SVM: support vector machine
XGBoost: extreme gradient boosting
