# Supplementary material for: Performance Drift in Machine Learning Models for Cardiac Surgery Risk Prediction: Retrospective Analysis
Source: JMIRx Med. 2024 Jun 12;5:e45973. doi: 10.2196/45973 (PMC11217160; doi:10.2196/45973)
Supplement: Multimedia Appendix 1 [file xmed-v5-e45973-s001.docx]

# **Section 1: Data Set Split**

## **Section 1.1: Characteristics of Training/Validation and Holdout cohorts**

Table S1. Distribution of cardiac surgical procedures for Training/Validation Data (years 2012-2016); WOI - Weight of intervention.

|  | **Operative mortality** | |  |
| --- | --- | --- | --- |
| **Variable** | **No, N = 152,819^1^** | **Yes, N = 4,377^1^** | **P value^2^** |
| Age (years), mean (SD) | 69.32 (8.20) | 72.34 (8.45) | <0.001 |
| Female gender, n (%) | 41,474 (27%) | 1,639 (37%) | <0.001 |
| Operative urgency (ES II), n (%) |  |  | <0.001 |
| 1 – Elective | 99,722 (65%) | 1,772 (40%) |  |
| 2 – Urgent | 48,394 (32%) | 1,489 (34%) |  |
| 3 – Emergency | 4,308 (2.8%) | 815 (19%) |  |
| 4 – Salvage | 363 (0.2%) | 300 (6.9%) |  |
| Unknown | 32 | 1 |  |
| Diabetes on insulin, n (%) | 8,808 (5.8%) | 319 (7.3%) | <0.001 |
| Year of Procedure, mean (SD) |  |  | <0.001 |
| 2012 | 30,723 (20%) | 974 (22%) |  |
| 2013 | 30,345 (20%) | 899 (21%) |  |
| 2014 | 30,766 (20%) | 877 (20%) |  |
| 2015 | 30,223 (20%) | 808 (18%) |  |
| 2016 | 30,762 (20%) | 819 (19%) |  |
| Chronic pulmonary disease, n (%) | 18,008 (12%) | 821 (19%) | <0.001 |
| Extracardiac arteriopathy, n (%) | 16,155 (11%) | 869 (20%) | <0.001 |
| Previous cardiac surgery, n (%) | 8,494 (5.6%) | 815 (19%) | <0.001 |
| Surgery on thoracic aorta, n (%) | 5,966 (3.9%) | 595 (14%) | <0.001 |
| Active endocarditis, n (%) | 3,742 (2.4%) | 336 (7.7%) | <0.001 |
| Critical preoperative state, n (%) | 5,061 (3.3%) | 946 (22%) | <0.001 |
| Renal impairment, n (%) |  |  | <0.001 |
| 0 - Normal | 16,664 (11%) | 1,278 (29%) |  |
| 1 - Moderate | 63,667 (42%) | 1,718 (39%) |  |
| 2 - On Dialysis | 70,991 (46%) | 1,148 (26%) |  |
| 3 - Severe | 1,497 (1.0%) | 233 (5.3%) |  |
| Recent myocardial infarction, n (%) | 29,722 (19%) | 1,104 (25%) | <0.001 |
| NYHA, n (%) |  |  | <0.001 |
| 0 – I | 33,559 (22%) | 725 (17%) |  |
| 1 – II | 67,146 (44%) | 1,130 (26%) |  |
| 2 – III | 44,357 (29%) | 1,598 (37%) |  |
| 3 – IV | 7,757 (5.1%) | 924 (21%) |  |
| CCS class 4 angina, n (%) | 12,583 (8.2%) | 646 (15%) | <0.001 |
| Neurological dysfunction/Poor mobility, n (%) | 4,934 (3.2%) | 302 (6.9%) | <0.001 |
| LVEF (ES II), n (%) |  |  | <0.001 |
| 0 - Good (>50%) | 863 (0.6%) | 106 (2.4%) |  |
| 1 - Moderate (31-50%) | 2,761 (1.8%) | 200 (4.6%) |  |
| 2 - Poor (21-30%) | 19,554 (13%) | 710 (16%) |  |
| 3 - Very Poor (≤20%) | 129,641 (85%) | 3,361 (77%) |  |
| Systolic pulmonary pressure (ES II), n (%) |  |  | <0.001 |
| 0 – PA Systolic (<31mmHg) | 139,360 (91%) | 3,481 (80%) |  |
| 1 – PA Systolic (31-55 mmHg) | 9,395 (6.1%) | 496 (11%) |  |
| 2 – PA Systolic (>55mmHg) | 4,064 (2.7%) | 400 (9.1%) |  |
| WOI Single Non-CABG, n (%) | 42,552 (28%) | 1,475 (34%) | <0.001 |
| WOI 2 Procedures, n (%) | 29,256 (19%) | 1,487 (34%) | <0.001 |
| WOI 3 Procedures, n (%) | 2,993 (2.0%) | 320 (7.3%) | <0.001 |
| ^1^Mean (SD) or Frequency (%) | | | |
| ^2^Wilcoxon rank sum test; Pearson's Chi-squared test or Fisher’s exact test | | | |

Table S2. Distribution of cardiac surgical procedures for Holdout Data (years 2017-2019); WOI -	Weight of intervention.

|  | **Operative mortality** | |  |
| --- | --- | --- | --- |
| **Variable** | **No, N = 68,010^1^** | **Yes, N = 1,881^1^** | **P value^2^** |
| Age (years), mean (SD) | 69.14 (8.05) | 71.76 (8.39) | <0.001 |
| Female gender, n (%) | 17,993 (26%) | 689 (37%) | <0.001 |
| Procedure urgency (ES II), n (%) |  |  | <0.001 |
| 1 – Elective | 41,820 (62%) | 665 (35%) |  |
| 2 – Urgent | 23,696 (35%) | 645 (34%) |  |
| 3 – Emergency | 2,225 (3.3%) | 415 (22%) |  |
| 4 – Salvage | 226 (0.3%) | 152 (8.1%) |  |
| Unknown | 43 | 4 |  |
| Diabetes on insulin, n (%) | 4,010 (5.9%) | 134 (7.1%) | 0.026 |
| Year of Procedure, mean (SD) |  |  | 0.692 |
| 2017 | 31,499 (46%) | 890 (47%) |  |
| 2018 | 29,489 (43%) | 800 (43%) |  |
| 2019 | 7,022 (10%) | 191 (10%) |  |
| Chronic pulmonary disease, n (%) | 8,636 (13%) | 390 (21%) | <0.001 |
| Extracardiac arteriopathy, n (%) | 6,172 (9.1%) | 346 (18%) | <0.001 |
| Previous cardiac surgery, n (%) | 3,518 (5.2%) | 326 (17%) | <0.001 |
| Surgery on thoracic aorta, n (%) | 3,104 (4.6%) | 301 (16%) | <0.001 |
| Active endocarditis, n (%) | 2,074 (3.0%) | 157 (8.3%) | <0.001 |
| Critical preoperative state, n (%) | 2,194 (3.2%) | 436 (23%) | <0.001 |
| Renal impairment, n (%) |  |  | <0.001 |
| 0 - Normal | 6,371 (9.4%) | 495 (26%) |  |
| 1 - Moderate | 28,744 (42%) | 733 (39%) |  |
| 2 - On Dialysis | 32,205 (47%) | 556 (30%) |  |
| 3 - Severe | 690 (1.0%) | 97 (5.2%) |  |
| Recent myocardial infarction, n (%) | 13,594 (20%) | 490 (26%) | <0.001 |
| NYHA, n (%) |  |  | <0.001 |
| 0 – I | 15,066 (22%) | 330 (18%) |  |
| 1 – II | 29,742 (44%) | 479 (25%) |  |
| 2 – III | 19,692 (29%) | 630 (33%) |  |
| 3 – IV | 3,510 (5.2%) | 442 (23%) |  |
| CCS class 4 angina, n (%) | 5,787 (8.5%) | 310 (16%) | <0.001 |
| Neurological dysfunction/Poor mobility, n (%) | 3,371 (5.0%) | 212 (11%) | <0.001 |
| LVEF (ES II), n (%) |  |  | <0.001 |
| 0 - Good (>50%) | 396 (0.6%) | 39 (2.1%) |  |
| 1 - Moderate (31-50%) | 1,480 (2.2%) | 118 (6.3%) |  |
| 2 - Poor (21-30%) | 11,054 (16%) | 379 (20%) |  |
| 3 - Very Poor (≤20%) | 55,080 (81%) | 1,345 (72%) |  |
| Systolic pulmonary pressure (ES II), n (%) |  |  | <0.001 |
| 0 – PA Systolic (<31mmHg) | 62,283 (92%) | 1,519 (81%) |  |
| 1 – PA Systolic (31-55 mmHg) | 3,731 (5.5%) | 209 (11%) |  |
| 2 – PA Systolic (>55mmHg) | 1,996 (2.9%) | 153 (8.1%) |  |
| WOI Single Non-CABG, n (%) | 20,016 (29%) | 678 (36%) | <0.001 |
| WOI 2 Procedures, n (%) | 13,393 (20%) | 621 (33%) | <0.001 |
| WOI 3 Procedures, n (%) | 1,376 (2.0%) | 131 (7.0%) | <0.001 |
| ^1^Mean (SD) or Frequency (%) | | | |
| ^2^Wilcoxon rank sum test; Pearson's Chi-squared test; Fisher's exact test | | | |

## **Section 1.2: Variables used for ES II**

| Table S3. ES II variables included in ML models; score coefficients and constants used for ES II score calculation are shown; these are not used for ML models; bold variables in ES II are substantially different to the earlier version Logistic EuroSCORE; | | | | |
| --- | --- | --- | --- | --- |
| **ES II** | **Coefficient** | |  |  |
| Age | 0.0285181 | |  |  |
| Female gender | 0.2196434 | |  |  |
| **Renal impairment**  -          On dialysis  -          CrCl≤50 (Severe)  -          CrCl 50-85 (Moderate) | ≡  0.6421508  0.8592256  0.303553 | |  |  |
| Extracardiac arteriopathy | 0.5360268 | |  |  |
| Chronic pulmonary disease | 0.1886564 | |  |  |
| Neurological dysfunction (Poor mobility) | 0.2407181 | |  |  |
| Previous cardiac surgery | 1.118599 | |  |  |
| Recent myocardial infarction | 0.1528943 | |  |  |
| **Left ventricular ejection fraction (LVEF)**  -          Moderate (31-50%)  -          Poor (21-30%)  -          Very Poor (≤20%) | ≡  0.3150652  0.8084096  0.9346919 | |  |  |
| **Systolic pulmonary pressure**  -          31-55 mmHg  -          ≥55mm Hg | ≡  0.1788899  0.3491475 | |  |  |
| Active endocarditis | 0.6194522 | |  |  |
| CCS class 4 angina | 0.2226147 | |  |  |
| **Operative urgency**  -          Urgent  -          Emergency  -          Salvage | ≡  0.3174673  0.7039121  1.362947 | |  |  |
| Critical preoperative state | 1.086517 | |  |  |
| - |  | |  |  |
| **Weight of intervention**  -          Single Non-CABG  -          2 Procedures  -          3 Procedures | ≡  0.0062118  0.5521478  0.9724533 | |  |  |
| Surgery on thoracic aorta | 0.6527205 | |  |  |
| **NYHA**  -          II  -          III  -          IV | 0.1070545  0.2958358  0.5597929 | |  |  |
| **Diabetes on insulin** | 0.3542749 | |  |  |
|  |  |  | |  |

**βo**  constant	−5.324537

# **Section 2: Model Specification**

Neural Network (Neuronetwork) was trained using 1000 epochs, with batch size of 20,000. The 2012–2016 dataset was split 70:30, with 70% used as training data and 30% as validation data for early stopping to reduce likelihood of overfitting [22]. The best model was saved using early stopping to prevent overfitting [23]. Binary cross-entropy loss was used as the loss function, with Adam as the optimizer [24], monitoring on accuracy as the metric. The final model configuration used for evaluation was the optimal set derived from our previous study on the NACSA Bristol cohort: input layer n = 18 nodes, hidden layer one n = 90 nodes, hidden layer two n = 36 nodes and output layer one node [19].

For the new models Weighted SVM and Xgboost, for which optimal parameters have not been investigated in our previous study [19], 3-fold Grid Search Cross Validation was applied using 2012-2016 dataset to determine the optimal hyperparameters to apply to 2017–2019 test dataset [25]. For Random Forest, the final model configuration used for evaluation was the optimal set derived from our previous study on the NACSA Bristol cohort [19]. This consisted of manually tuned parameters in response to model discrimination (AUC) evaluated with cross-validation (estimators n = 700, maximum depth n = 10, minimum samples split n = 5, minimum samples leaf n = 20) [19]. The ES II risk factors were fitted with an LR (retrained LR) model with Inverse of regularization strength (C) set to 1 [19].

Seed

Number = 7 for reproducibility of results.

Optimal hyperparameters

*Xgboost*

{'max_depth': 5, 'eta': 1, 'objective': 'binary:logistic', 'subsample': 0.6, 'min_child_weight': 1, 'gamma': 0.5, 'colsample_bytree': 0.8, 'learning_rate' : 0.02, 'n_estimators' : 300}

**Optimal Model Selection**

Table S4. Anova Test for difference across all six models including ES II; ges - generalized eta squared is a measure of effect size

Effect	DFn	DFd	F	p	p<.05	ges

1	Model	5	5994	221615.2	0	*	0.995

**Consort Diagram**

Figure S1. Consort diagram showing flow of participants through the study.

**Baseline non-temporal performance**

Figure S2. models are compared against each other using multiple pairwise paired t-tests.

**Drift Analysis**

Figure S3. Clinical Effectiveness Metric (CEM) overall performance drift by year month; linear regression lines are plotted for each model with slope, intercept and p-values displayed in legend; SVM and ES II are removed to enable clearer separation of models with similar performance.

Figure S4. Clinical Effectiveness Metric (CEM) overall performance drift by year month; linear regression lines are plotted for each model with slope, intercept and p-values displayed in legend.

Figure S5. Linear regression diagnostic plots for models (Xgboost; Random Forest; Logistic Regression; EuroSCORE II): left-column QQ plot of residuals to check normality assumptions for linear regression (lm) of model CEM against year month (Ym); right-column: scale-location plot for homogeneity of residual variance (homoscedasticity); fitted values of the outcome variable against square root of standardised residual points; year month (Ym); linear regression (lm).

Table S5. Differences across models for the first 3 months of 2017 were tested using Kruskal-Wallis Test; chi-squared statistic and p-values are shown.

Metric	n	statistic	df	p	method

1	CEM	18000	12326	5	0	Kruskal-Wallis

Figure S6. Paired samples Wilcoxon test (Wilcoxon signed-rank test) for first 3 months of 2017 bootstrap CEM values; p-values are adjusted using the bonferroni method.

Table S6. Dunn test for non-parametric multiple comparison of models for the first three months of 2017; median CEM and difference of median CEM to Xgboost baseline are shown; non-adjusted and bonferroni adjusted p-values are shown for Dunn test.

| Model | Baseline | Median CEM | Diff Xgboost | n1 | n2 | p | p.adj | p.adj.signif |
| --- | --- | --- | --- | --- | --- | --- | --- | --- |
| Random Forest | Xgboost | 0.737 | -0.0076 | 3000 | 3000 | 3.32E-07 | 4.97E-06 | **** |
| Neuronetwork |  | 0.732 | -0.0124 | 3000 | 3000 | 1.01E-23 | 1.01E-23 | **** |
| Logistic Regression |  | 0.731 | -0.0138 | 3000 | 3000 | 1.35E-26 | 2.02E-25 | **** |
| Weighted SVM |  | 0.648 | -0.0961 | 3000 | 3000 | 0.00E+00 | 0.00E+00 | **** |
| EuroSCORE II |  | 0.471 | -0.2739 | 3000 | 3000 | 0.00E+00 | 0.00E+00 | **** |

Table S7. Differences across models for the first 3 months of 2019 were tested using Kruskal-Wallis Test; chi-squared statistic and p-values are shown.

Metric	n	statistic	df	p	method

1	CEM	18000	12041	5	0	Kruskal-Wallis

Table S8. Dunn test for non-parametric multiple comparison of models for the first three months of 2019; median CEM and difference of median CEM to Xgboost baseline are shown; non-adjusted and bonferroni adjusted p-values are shown for Dunn test.

| Model | Baseline | Median CEM | Diff Xgboost | n1 | n2 | p | p.adj | p.adj.signif |
| --- | --- | --- | --- | --- | --- | --- | --- | --- |
| Random Forest | Xgboost | 0.713 | -0.0032 | 3000 | 3000 | 3.22E-03 | 4.82E-02 | * |
| Logistic Regression |  | 0.710 | -0.0055 | 3000 | 3000 | 2.13E-06 | 3.19E-05 | **** |
| Neuronetwork |  | 0.705 | -0.0108 | 3000 | 3000 | 1.91E-20 | 2.87E-19 | **** |
| Weighted SVM |  | 0.630 | -0.0856 | 3000 | 3000 | 0.00E+00 | 0.00E+00 | **** |
| EuroSCORE II |  | 0.456 | -0.2594 | 3000 | 3000 | 0.00E+00 | 0.00E+00 | **** |

Table S9. Differences of models’ CEM across the first 3 months of 2017 and 2019 were tested using Kruskal-Wallis Test; chi-squared statistic and p-values are shown.

Metric	n	statistic	df	p	method

1	CEM	36000	1264	1	6.18e-277	Kruskal-Wallis

Figure S7. Kruskal-Wallis Test and paired samples Wilcoxon test (Wilcoxon signed-rank test) to test for difference in models’ CEM across first three months of 2017 and 2019; Bonferroni correction is not applied as there are only two comparisons.

Table S10. Dunn test for non-parametric multiple comparison of difference in model CEM across the first three months of 2017 and 2019; median (Med) CEM and difference (Diff) across two time points are shown; non-adjusted and bonferroni adjusted p-values are shown for Dunn test.

| Model | Med 2017 | Med 2019 | Med Diff | statistic | p | p.adj | p.adj.signif |
| --- | --- | --- | --- | --- | --- | --- | --- |
| Xgboost | 0.744 | 0.716 | 0.0288 | -20.94 | 2.21e-97 | 1.46e-95 | **** |
| Random Forest | 0.737 | 0.713 | 0.0244 | -20.01 | 4.75e-89 | 3.14e-87 | **** |
| Neuronetwork | 0.732 | 0.705 | 0.0272 | -20.45 | 5.77e-93 | 3.80e-91 | **** |
| Logistic Regression | 0.731 | 0.710 | 0.0205 | -16.26 | 1.92e-59 | 1.27e-57 | **** |
| Weighted SVM | 0.648 | 0.630 | 0.0183 | -5.92 | 3.21e-09 | 2.18e-07 | **** |
| EuroSCORE II | 0.471 | 0.456 | 0.0142 | -3.52 | 4.34e-04 | 2.87e-02 | * |

Figure S8. Linear regression diagnostic plots for models (Logistic Regression; Neuronetwork; Random Forest; EuroSCORE II; Weighted SVM; Xgboost): left-column QQ plot of residuals to check normality assumptions for linear regression (lm) of model AUC against year month (Ym); right-column: scale-location plot for homogeneity of residual variance (homoscedasticity); fitted values of the outcome variable against square root of standardised residual points.

## Positive outcome discrimination

Linear regression plots show that Xgboost has the best F1 throughout the Holdout period (intercept = 0.335 vs. 0.334 (RF), 0.314 (LR), 0.313 (NN) ). Rate of performance decrease for Xgboost was less than RF (slope: -0.00113 vs. -0.00117; Figure S9), but greater than all other models. Although SVM, LR and NN showed lower rates of F1 decrease (slope: -0.000756, -0.000968, -0.00101), the absolute F1 score across Holdout time periods was substantially lower than Xgboost and RF. The best performing model across all Holdout time periods was Xgboost, followed by RF, LR, NN, SVM and then ES II. There was a strong evidence of decrease in F1 performance across all models (p < 0.0001). Normality and homogeneity assumptions were satisfied for all model F1 values as checked by QQ plot of residuals and scale-location plot (Figure S10).

Figure S9. F1 performance drift by year month; linear regression lines are plotted for each model with slope, intercept and p-values displayed in legend.

Figure S10. Linear regression diagnostic plots for models (Logistic Regression; Neuronetwork; Random Forest; EuroSCORE II; Weighted SVM; Xgboost): left-column QQ plot of residuals to check normality assumptions for linear regression (lm) of model F1 against year month (Ym); right-column: scale-location plot for homogeneity of residual variance (homoscedasticity); fitted values of the outcome variable against square root of standardised residual points.

Figure S11. Calibration (ECE) performance drift by year month; linear regression lines are plotted for each model with slope, intercept and p-values displayed in legend.

Figure S12. Linear regression diagnostic plots for models (Logistic Regression; Neuronetwork; Random Forest; EuroSCORE II; Weighted SVM; Xgboost): left-column QQ plot of residuals to check normality assumptions for linear regression (lm) of model ECE against year month (Ym); right-column: scale-location plot for homogeneity of residual variance (homoscedasticity); fitted values of the outcome variable against square root of standardised residual points.

Figure S13. Clinical effectiveness (net benefit) performance drift by year month; linear regression lines are plotted for each model with slope, intercept and p-values displayed in legend.

Figure S14. Linear regression diagnostic plots for models (Logistic Regression; Neuronetwork; Random Forest; EuroSCORE II; Weighted SVM; Xgboost): left-column QQ plot of residuals to check normality assumptions for linear regression (lm) of model net benefit against year month (Ym); right-column: scale-location plot for homogeneity of residual variance (homoscedasticity); fitted values of the outcome variable against square root of standardised residual points.

## Accuracy of prediction probability

Linear regression plots show that Xgboost has the best adjusted Brier performance throughout the Holdout period (intercept: 0.9764, slope: 2.96e-6; Figure S15). Although LR had better starting Brier (0.9763), a high rate of decrease (slope: -1.1e-5 vs. 9.12e-6) resulted RF being the second best model by march 2019, followed by LR and then NN. ES II performed worst in terms of Brier and rate of decrease, followed by SVM (Figure S16). There was a strong evidence of decrease in Brier performance across all models (p < 0.0001), except Xgboost and RF, whose gradients were positive. Normality and homogeneity assumptions were satisfied for all model Brier (adjusted) values as checked by QQ plot of residuals and scale-location plot (Figure S17).

Figure S15. Adjusted Brier (1-Brier) performance drift by year month; linear regression lines are plotted for each model with slope, intercept an	d p-values displayed in legend; SVM and ES II are removed to enable clearer separation of models with similar performance.

Figure S16. Adjusted Brier (1-Brier) performance drift by year month; linear regression lines are plotted for each model with slope, intercept and p-values displayed in legend.

Figure S17. Linear regression diagnostic plots for models (Logistic Regression; Neuronetwork; Random Forest; EuroSCORE II; Weighted SVM; Xgboost): left-column QQ plot of residuals to check normality assumptions for linear regression (lm) of model Brier against year month (Ym); right-column: scale-location plot for homogeneity of residual variance (homoscedasticity); fitted values of the outcome variable against square root of standardised residual points.

Figure S18. Age dataset drift across year month for Holdout set; percentages of each category are shown for each time point; age values are binned into those above 60 and those 60 and below.

Figure S19. Weight of intervention dataset drift across year month for Holdout set; percentages of each category are shown for each time point.
